# Transactions of the Homœopathic Medical Society of Pennsylvania

**Published:** 1896-04

**Authors:** 


TRANSACTIONS OF THE THIRTY-FIRST SESSION OF THE HOMŒOPATHIC MEDICAL SOCIETY OF THE STATE OF PENNSYLVANIA, held at Pittsburg, September 17th, 18th, 19th, 1895. Philadelphia: Sherman & Co., printers, 1896.

It is not possible for the editor of this journal to discuss the transactions of societies. There is too much and in too great variety in these volumes for any ordinary review. It will, therefore, suffice to say that the volume before us is highly interesting and instructive. Among the articles deserving of special notice may be mentioned "Galactacrasia," by Dr. Pearl Starr; "The Chloroform and Oxygen Combination as an Anæsthetic," by H. L. Northrop, M. D.; "The New Anæsthetic—Oxygenated Chloroform," by Dr. W. H. Cooper; "The Proper Application of the Homœopathic Materia Medica," by Dr. J. C. Guernsey; "Splinter-like Pains Compared; or Keynote Modulations," by Dr. Edward Cranch. This last should be carefully studied by every "symptom coverer" and entered in a manuscript repertory alphabetically arranged as directed by Dr. Yingling in his article on "Private Repertories," published in THE HOMŒOPATHIC PHYSICIAN for March, 1891, page 124. "Optimism *vs.* Pessimism in the Evolution of our Materia Medica," by Dr. Roland T. White. "A Plea for Purer Practice," by Dr. Z. T. Miller.

There are many more of these valuable articles not mentioned in this notice, but enough have been enumerated to show the value of the new volume of the State Society's work.

